# Exploring leadership behaviors of the coaches of champion teams

**DOI:** 10.3389/fpsyg.2022.1091703

**Published:** 2023-01-04

**Authors:** Eda Yenen, Hakan Atamturk, Nurdan Atamturk

**Affiliations:** ^1^Department of Recreation, Near East University, Nicosia, Cyprus; ^2^Department of English Language Teaching, University of Kyrenia, Kyrenia, Cyprus

**Keywords:** student athletes, leadership, coach leadership, sport psychology, type of sport

## Abstract

Participation in sports at school is highly valued since it is believed that it develops students’ social skills and adds to personal development. Owing to the fact that the behaviors of the coaches have an important effect on the development of the student athletes, this quantitative study was designed with the aim to evaluate the perceptions of young student athletes of the champion school teams of their coaches’ leadership behaviors. The study also sought to determine the variables affecting these perceptions. A total of 236 young student athletes who were the members of the champion teams of basketball, handball, volleyball, futsal, badminton, wrestling and swimming in the period of 2021–2022 evaluated their coaches’ leadership qualities. One of the major results was that the young student athletes had positive perceptions of their coaches. Adopting Chelladurai’s model, training and instruction was found to be particularly important in achieving high performance. More specifically, the Mann Whitney *U* test results revealed that the athletes’ perceptions differed by gender and by being involved in team or individual sports; however, Kruskal-Wallis test revealed perceptions did not differ by age. The significance of this study lies in its practical implications for sport coaches, coaching practice, physical education teachers and practitioners in sport psychology.

## Introduction

Leadership is a concept as old as human history. As well as being social creatures living in groups, individuals are beings who need hierarchical commitment with the leader who will manage the groups they create and lead them to their goals (Nacar and Gacar, 2013). Studies to reveal group dynamics in sports have focused on many aspects of the group, namely group size, structure, leadership, cohesion, motivation, task, intimacy and competence. Leadership, which is viewed as one of them, is the combination of characteristics that direct people toward a desired goal and is required to lead and manage a particular community. In this respect, leadership can be defined as a behavioral process that affects individuals and groups for specified purposes (Barrow, 1977).

Research in the relevant field indicates that the “effective coach” is the leader who achieves successful performance. An effective coach must be prepared to respond to various personal and individual needs of athletes. The effective coach can make a difference in the performance of the team by improving his coaching skills and knows the effect of his own behavior on the athletes (Anshel, 2003).

Leadership characteristics and behaviors of the coach have become the most researched subject in recent years. In order to achieve success, a coach must motivate his players, increase morale and give an impetus to their work. For this reason, the coach has to show leader behaviors. Drawing on this notion, it can be suggested that coaches are highly associated with leaders (Barrow, 1977). Besides, it has been observed that coach behaviors have a significant effect on athlete development (Smoll and Smith, 1984). In terms of coaches, leadership firstly manifests itself in guiding and directing athletes (İkizler, 2000). Studies have shown that an effective leader has a significant contribution to the success of the team, to establish the team spirit or to reach the intended goal for the athletes or many others who are interested in sports (Rayburn et al., 2001).

Young student athletes represent their schools and compete for the success of their school teams. Such competitions among schools put extra effort to young student athletes since they have to train very long hours after school with their coaches and they have to establish a good rapport with their coaches. In order to be successful as a team, coaches need to be very careful to develop young student athletes’ physical and technical capabilities to foster sports performance (Cruz and Kim, 2017). Despite a plethora of research studies on leadership worldwide, there is only a handful of studies on leadership in sports in the Turkish context and to the best of our knowledge there is none in the Turkish Cypriot context. The rationale behind the design of this study is that most studies in the relevant literature are conducted with the athletes of one or two sport branches. To address this void, this study comprises seven sports branches, namely basketball, handball, volleyball, futsal, badminton, wrestling and swimming. Additionally, the participants of a great majority of research studies in the literature are adult professional athletes competing in professional leagues; however, the participants of this study are secondary school student athletes competing in the school sports leagues. Last but not least, leadership research is dominated by the perceptions of athletes of their coaches (Sullivan and Kent, 2003; Tojjari et al., 2013; Szedlak et al., 2015; González-García et al., 2019; Keatlholetswe and Malete, 2019) and the role of effective leadership on athletes’ performance (Myers et al., 2006; Horn, 2008; Kavussanu et al., 2008; Szedlak et al., 2015). While this is the case, Keatlholetswe and Malete (2019) argue that more studies which incorporate athletes’ evaluations of coaches’ leadership styles still need to be conducted to get a broader perspective. In this respect, they suggest a shift from the perspective of the coach to the perspective of the athlete. Drawing on this argument, the current study was designed to build on the previous knowledge regarding athletes’ perceptions of their coaches and to investigate athletes’ preferences and beliefs and the effect of athletes’ demographic variables on the perceptions of the leadership behaviors of their coaches. While previous studies examined the effect of such demographic variables as gender and age, few studies examined the likely effects of the type of sports (individual\team) which athletes are involved in. Neither did they examine the effect of demographic variables, such as gender on the perceptions of the athletes of team and individual sports. We wanted to examine these relationships with the athletes of champion teams of a variety of sport branches. Since all the participating teams were champion teams and that effective leadership was associated with success as evidenced by the previous research, it was hypothesized that athletes would have positive perceptions of their coaches’ leadership qualities. Additionally, based on the theoretical framework posed by Chelladurai and Saleh (1980) as well as the previous research results, we hypothesized that training and instruction would be the mostly preferred leadership behavior.

### Theoretical underpinnings

Chelladurai and Saleh’s (1980) multidimensional model of coach leadership was preferred as the theoretical framework in the present study. The rationale behind this preference was that this theoretical model specifically focuses on sport contexts and includes many varieties of coach leadership behaviors that best fit a number of sport modalities. Since we aimed to investigate athletes perceptions of their coaches, it was thought that Chelladurai and Saleh’s (1980) multidimensional model would work well in the context of the study.

### Aim of the study

Given that coaches are important in young athletes’ lives and that effective coaches have to possess leadership qualities, this quantitative study was designed with the aim to evaluate the leadership qualities of the coaches of champion teams from the viewpoint of young student athletes. Additionally, the independent variables affecting the perceptions of the young student athletes of the leadership characteristics of their coaches were also determined.

### Research questions

In order to fulfill the research aim the following questions were posed:

1. How do the young student athletes of champion teams perceive the leadership qualities of their coaches?
2. To what extent do the perceptions of the participants differ by gender, age and type of sport (individual or team)?

## Review of literature

Considering all models developed in the field of leadership, the most frequently used and tested model by researchers is Chelladurai’s (1993, 2007), multidimensional leadership model. An effective coach is a person who is prepared to respond to the personal expectations of the athletes, is aware of the fact that he can make a difference in the performance of the team by improving his coaching skills, and knows the effect of his own behavior on the athletes. An effective coach is also a good leader. Researchers have adopted the notion that effective leadership and coaching is a function of situational and individual characteristics (Chelladurai and Riemer, 1998).

Leadership in sports has gained importance due to the establishment of sports on scientific foundations. The leadership function of the coach is listed among the most important duties of the coach in various sources. In team games, in order to ensure team integrity and success, a coach with leadership characteristics and athletes who will work for this success are needed. Being the leaders of sports teams, coaches need to display various leadership behaviors. These behaviors have a very important role in the team’s achievement of the goals and meeting the needs of the team members (Donuk, 2006). Ultimately, what determines effective coach behavior is the individual qualities and the function of individual qualities. At the same time, effective coach behavior can reveal the athlete’s sense of achievement.

Schruijer and Vansina (2002) have found that establishing quality relationships between the coach and athletes is essential to effective and successful leadership. Given that the coach and athletes have a mutual relationship in their struggle toward their goal, the quality of relationships between them makes the leadership qualities of the coach an important research topic (Jowett and Chaundy, 2004). Effective leaders with essential leader qualities are needed to develop quality relations with athletes so that athletes can grow commitment and enthusiasm.

In the Turkish context, Nacar (2013) has investigated the leadership styles of the coaches working in the Turkish professional handball league to find that there is a statistically significant difference between the views of the female and male handball players of their coaches’ training and instruction behavior. However, Nacar’s (2013) study has not revealed a statistically significant difference in terms of the autocratic and social support behavior. Nacar and Gacar (2013) have examined coach-leader relationship and team unity in volleyball. This study has revealed that while social support behavior differed by gender, participant perceptions of the coaches’ training and instruction, democratic, autocratic and positive feedback behavior did not.

Fletcher and Roberts (2013) have found that the participating athletes perceived autocratic behavior to be the least-frequent leadership behavior displayed by their coach. In a similar vein, Sherman et al. (2000) have reported congruent results that female athletes prefer autocratic behavior to be the least-frequent leadership behavior. Further, Riemer and Toon (2001) evaluate the effect of gender on the perceptions of leadership to find that female athletes perceived low levels of autocratic behavior. Sherman et al. (2000) also report similar findings that females perceived less autocratic behavior. Australian athletes prefer a democratic decision-making style (Sherman et al., 2000), which is also preferred by coaches (Weinberg and McDermott, 2002). Fletcher and Roberts’ (2013) study also reports high perceptions of female athletes of the sub-dimensions of training and instruction and positive feedback behavior. This result is endorsed by those of previous studies that female athletes prefer training and instruction and positive feedback behavior (Riemer and Toon, 2001; Witte, 2011).

## Materials and methods

### Research model

A quantitative research design and survey method was utilized in this study which aimed to evaluate the perceptions of the young student athletes of the leadership behavior characteristics of their coaches of the champion teams of the basketball, handball, volleyball, futsal, badminton, wrestling and swimming in North Cyprus.

### Subjects

Eight basketball teams, 10 handball teams, 8 volleyball teams, 6 futsal teams, 7 badminton teams, 8 wrestling teams and 12 swimming teams competed in the school sports national leagues organized by the Ministry of National Education and Culture in North Cyprus in the period of 2021–2022. Only the champion school teams of each sports branch during this period were included in this study. Overall, 236 young student athletes who were the members of the champion teams of basketball, handball, volleyball, futsal, badminton, wrestling and swimming participated in the study. The age range of the participants was 9–10 (*n* = 14), 11–12 (*n* = 40), 13–14 (*n* = 36), 15–16 (*n* = 54), 17–18 (*n* = 28), 19 and over (*n* = 64). Eighty three participants were female and a 153 of them were male. Participation was on a voluntary basis and an informed consent form was filled out and signed by the participants who were 18 and above and by the parents whose children were under 18. The study was conducted according to international ethical guidelines. An ethical consent was obtained from a higher institution review board.

### Data collection

As the data collection instrument Chelladurai and Saleh’s (1980) Leadership Scale for Sports (LSS) was utilized since this study focused on how athletes perceived their coaches’ leadership style. LSS contained 40 items to assess leadership behavior and evaluate the likely relationships within the Multidimensional Model of Leadership (MML). LSS consists of five subscales, namely democratic, autocratic, training, and instruction, social support, and positive feedback. Each of these subscales stands for a dimension of leadership behavior. The first subscale which is democratic behavior is evaluated through nine items in the instrument and evaluates whether or not the coach includes the athletes in the decision making and goals setting procedures. Five items are allocated to the autocratic behavior subscale which reflects the decision style of the coach and evaluates the relationship between the coach and the athletes in terms of closeness. Thirteen items evaluate training and instruction representing the extent to which the coach enables the athletes to develop skills and help learn the tactics. Additionally, by structuring and coordinating the activities of team members, it clarifies the relationships between them. Eight items of the scale are allotted to social support behavior which refers to the coach’s ability to create a supportive environment among the athletes to satisfy the interpersonal needs of the athletes. Five items evaluate positive feedback behavior which refers to the coach’s ability to acknowledge and appreciate the athlete’s efforts and desirable performance. Responses were elicited *via* a five-point scale ranging from always (5) to never (1). Cronbach’s alphas for the subscales of democratic behavior, autocratic behavior, training and instruction, social support behavior, positive feedback behavior were found to be 0.87, 0.91, 0.79, 0.81, 0.91, respectively, which indicated the reliability as well as the internal consistency of the instrument.

### Data analysis

The distribution of the variables was tested with the Kolmogorov–Smirnov test and the Shapiro–Wilk test, and it was observed that all variables did not show normal distribution. For this reason, independent variables related to the problem and attitude scores were tested with a nonparametric test. The Mann–Whitney *U* test was performed for pairwise comparisons. *α* = 0.05 was chosen for the significance level.

### Reliability

Regarding the reliability of the instrument, the Cronbach’s alpha value for the reliability coefficients was found to be 0.91. The Cronbach’s alpha values of the dimensions were found to be 0.87 for training and instruction behavior, 0.86 for democratic behavior, 0.81 for social support behavior, 0.78 for positive feedback behavior and 0.75 for autocratic behavior. All Cronbach’s alpha values are over 0.74, which ensures the reliability of the instrument.

### Results

The means were interpreted according to the range and value of 4.20–5.00 = always, 3.40–4.19 = often, 2.60–3.39 = sometimes, 1.80–2.59 = rarely and 1.00–1.79 = never. The data analysis revealed that on average the mean scores of the leadership behavior of the coaches of the champion teams reflected responses of leader behavior higher than a response of *sometimes* which accounted for up to 84 % (see Table 1).

**Table 1:** Correlation Spearman’s rho, mean, standard deviation on the LSS (*n* = 236).

LSS	Training and instruction	Democratic behavior	Social support	Positive feedback	Autocratic behavior	Value
*M*	3.96	3.61	3.48	3.49	2.38	3.40–4.19 = often 1.80–2.59 = rarely
SD	0.63	0.79	0.64	0.71	0.68	

As Table 1 illustrated, training and instruction (*M* = 3.96, SD = 0.63) was the most frequently perceived leader behavior. Other perceived leader behaviors were democratic behavior (3.61, SD = 0.79), positive feedback (*M* = 3.49, SD = 0.71), social support (*M* = 3.48, SD = 0.64) and autocratic behavior (*M* = 2.38, SD = 0.68) respectively.

In reply to the second research question, it was found that the perceptions of the young athletes differed by gender and by being involved in team or individual sports but not by age.

### Gender

It was found that the perceptions of the participants differed by gender, *U* = 5958.500, *p* < 0.05 (see Table 2).

**Table 2:** Mann–Whitney *U* test results of the coach’s leadership behavior scores by gender.

Gender	*n*	Mean rank	Sum of ranks	*U*	*p*
Female	83	123.21	10226.50	5958.500	0.04
Male	153	105.94	17739.50		

As Table 2 illustrated, the mean scores of the perceptions of the female athletes of the coaches’ leadership behaviors were statistically higher than those of the male athletes.

### Differences by type of sports

The Mann–Whitney *U* test results indicated a significant difference between the perceptions of the athletes involved in team sports and individual sports in terms of the training and instruction behavior of the coach (*U* = 4947.00, *p* < 0.05; See Table 3).

**Table 3:** Mann–Whitney *U* test results concerning the training and instruction sub-dimension.

Team/Individual	*n*	Mean rank	Sum of ranks	*U*	*p*
Team	167	123.38	20604.00	4947.00	0.057
Individual	69	106.70	7362.00		

As Table 3 illustrated, it was found that the perceptions of the athletes of team sports of the training and instruction behavior of the coaches were higher than those of individual sports. Drawing on this statistically significant difference, we looked into how gender affected the perceptions of the athletes of team and individual sports. The Mann–Whitney *U* test results revealed a significant difference in the perceptions of leadership behaviors by gender (see Table 4).

**Table 4:** Mann–Whitney *U* test results by gender in team and individual sports.

Team/Individual	Gender	*n*	Mean rank	Sum of ranks	*U*	*p*
Team	Female	67	93.87	6289.50	3088.50	0.031
	Male	100	77.39	7738.50		
	Total	167				
Individual	Female	16	23.34	373.50	421.000	0.008
	Male	53	38.52	2041.50		
	Total	69				

Table 4 showed that the female athletes who were involved in team sports had more positive perceptions of their coaches and that the male athletes who were involved in individual sports had more positive perceptions of the coaches.

Concerning the effect of gender of the athletes involved in team and individual sports in terms of each of the five subscales of the LLS, the Mann–Whitney *U* test revealed that while there was no significant difference in training and instruction behavior and authoritarian behavior, significant differences were found in terms of democratic, social support and positive feedback behaviors.

The perceptions of the athletes involved in team or individual sports of their coaches’ democratic behaviors showed a significant difference by gender [*U*(2411.00) = 0.002, *p* < 0.05]. In team sports, the female athletes’ perceptions of the democratic behavior of their coaches were higher than the males. In this respect, it was found that the females in team sports and the males in individual sports perceived their coaches more positively in terms of democratic behaviors (see Table 5).

**Table 5:** Mann–Whitney *U* test results of the coach’s democratic behavior scores by gender.

Team/Individual	Gender	*n*	Mean rank	Sum of ranks	*U*	*p*
Team	Female	67	98.01	6567.00	2411.00	0.002
	Male	100	74.61	7461.00		
	Total	167				
Individual	Female	16	22.91	366.50	230.5	0.006
	Male	53	38.65	2048.50		
	Total	69				

The female athletes involved in team sports had more positive perceptions of their coaches’ democratic behavior than their male counterparts (*p* < 0.05). On the other hand, in terms of individual sports, the male athletes’ involved in individual sports had more positive perceptions of their coaches’ democratic behavior than the female athletes [*U*(230.5) = 0.006, *p* < 0.05].

Another statistically significant difference was found between the perceptions of the athletes involved in team or individual sports of their coaches’ social support behavior by gender (see Table 6).

**Table 6:** Mann–Whitney *U* test results of the coach’s scores on social support behaviors by gender.

Team/Individual	Gender	*n*	Mean rank	Sum of ranks	*U*	*p*
Team	Female	67	86.96	5826.00	3152.00	517.00
	Male	100	82.02	8202.00		
	Total	167				
Individual	Female	16	21.53	344.50	208.500	0.002
	Male	53	39.07	2070.50		
	Total	69				

The Mann–Whitney *U* test results revealed that the athletes’ perceptions of their coaches’ social support behaviors did not differ significantly by gender in team sports [*U* = (3152.00), *p* > 0.05]. Regarding individual sports, on the other hand, the perceptions of the male athletes’ perceptions of their coaches’ social support behaviors were found to be more positive than those of their female counterparts [*U* = (208.500), *p* < 0.05].

The other statistically significant difference was found between the perceptions of the athletes involved in team or individual sports of their coaches’ positive feedback behavior by gender (see Table 7).

**Table 7:** Mann–Whitney *U* test results of coaches’ scores on positive feedback behaviors by gender.

Team/Individual	Gender	*n*	Mean rank	Sum of ranks	*U*	*p*
Team	Female	67	100.34	6722.50	2255.50	0.00
	Male	100	73.06	7305.50		
	Total	167				
Individual	Female	16	18.38	294.00	158.00	0.00
	Male	53	40.02	2121.00		
	Total	69				

As Table 7 illustrated, when the team sports were considered, the perceptions of the female athletes of their coaches’ positive feedback behaviors were more positive than those of the male athletes [*U* = (2255.50), *p* < 0.05]. Similarly, when the individual sports were considered the perceptions of the athletes of their coaches’ positive feedback behaviors differed significantly by gender. When the perceptions of the female athletes of the positive feedback behaviors of their coaches (mean rank = 18.38) were compared with those of the male athletes (mean rank = 40.02), it was found that male athletes involved in team sports had more positive perceptions of their coaches’ positive feedback behaviors than the female athletes (*U* = (158.00), *p* < 0.05). As a result, the perceptions of the positive feedback (rewarding) behaviors of the coaches of the athletes involved in team and individual sports were found to be higher for women in team sports and men in individual sports.

### Age

Regarding age, it was found that the perceptions did not change by age (see Table 8).

**Table 8:** Kruskal-Wallis test results of the coach’s leadership behavior scores by age.

Age	*n*	Mean rank	SD	*X* ^2^	*p*
9–10	14	127.43	5	16.83	0.06
11–12	40	108.78			
13–14	36	97.65			
15–16	54	122.67			
17–18	28	106.43			
19>	64	112.68			

As Table 8 showed, there was not a statistically significant difference between the perceptions of the athletes by age [*X*^2^ (5) = 16.83, *p* > 0.05].

## Discussion

This study sought to investigate how the young student athletes of champion teams perceived the leadership qualities of their coaches as well as the variables which influence these perceptions. One of the major results of the current study was that the young student athletes of champion teams had positive perceptions of their coaches’ leadership qualities. It was an expected result since all the participating athletes were the members of the champion teams. Hence, due to the success of the teams, all coaches were expected to exhibit effective leadership skills. In this respect, the null hypothesis that the athletes of champion teams would have positive perceptions of their coaches’ leadership qualities was supported by this major result of this study. Chelladurai’s (1993, 2007), model draws parallels between athletes’ perceptions of specific types of coaching behavior and the effectiveness of coaching behavior. Concerning the effectiveness of the coach, the results indicated that training and instruction was the most frequently perceived leader behavior followed by democratic behavior, positive feedback, social support and autocratic behavior. With this result the second null hypothesis of the study that training and instruction would be the mostly preferred leadership behavior was also supported. This result indicated that the athletes considered the training and instruction behavior to be particularly important in achieving high performance. This result corroborated Sriboon’s (2001) argument that when coaches placed a high emphasis on training and instruction, the performance of sport teams increased significantly.

This result went in line with the results of previous studies which pointed out the benefits of leadership focused on training and instruction and positive feedback (Gillet et al., 2010; Weinberg and Gould, 2015; Cruz and Kim, 2017; Ekstrand et al., 2017; González-García et al., 2019; Koc, 2020). Training and instruction encompassed coaches’ skills to foster athletes’ performance in terms of skills, techniques and tactics. Thus, this result meant that the young athletes of this study found their coaches good at teaching them the required skills, techniques and tactics. This result also ensured that the young athletes acknowledged the effective role of their coaches in helping them establish healthy relationships among team members. The result was supported by the results of other studies in the literature (Chelladurai et al., 1988; Chelladurai and Riemer, 1998; Horn, 2008). It was also consistent with the results of previous studies (Sullivan and Kent, 2003; Hassani et al., 2013; Tojjari et al., 2013; Szedlak et al., 2015; Keatlholetswe and Malete, 2019). Szedlak et al. (2015) emphasized that although athletes in their study wanted their coaches to be caring and trustworthy, they valued the coaches’ instruction and technical knowledge more. This result could be acknowledged by Keatlholetswe and Malete’s (2019) argument that training and instruction, democratic leadership, and social support were highly valued in collectivist cultures (Hassani et al., 2013; Tojjari et al., 2013).

Moreover, the results indicated that the athletes’ perceptions differed by gender and type of sport (individual or team sport) but not by age. It was found in the current study that the female athletes perceived higher leadership behaviors of their coaches than their male counterparts. The result that gender as an independent variable influenced participants’ perceptions of leadership behaviors was consistent with the results of previous studies (Riemer and Toon, 2001). Aside from gender, the type of sports influenced the participants’ perceptions. The results indicated a significant difference between the perceptions of the athletes involved in team sports or individual sports in terms of the training and instruction behavior of the coach. More specifically, it was found that the athletes who were involved in team sports had more positive perceptions of the leadership qualities of their coaches in terms of training and instruction. Unfortunately, this result could not be supported due to the lack of studies on the issue.

Regarding how gender influenced the perceptions of leadership behavior by the type of sport, the results displayed that the female athletes who were involved in team sports had more positive perceptions of their coaches and that the male athletes who were involved in individual sports had more positive perceptions of the coaches. Owing to the lack of studies on the influence of the type of sport on the perceptions of leadership behaviors in the literature, this result could not be endorsed.

Moreover, the results indicated that gender influenced the perceptions of the athletes involved in individual and team sports in terms of democratic, social support and positive feedback behaviors but not training and instruction and authoritarian behaviors. The female athletes involved in team sports had more positive perceptions of their coaches’ democratic behavior than their male counterparts. Hence, although athletes generally appreciate coaches that enable and encourage their autonomy in decision-making within the training context (Weinberg and Gould, 2015; Cruz and Kim, 2017), the male athletes who were involved in team sports were not as positive as the females in team sports. As far as the individual sports were concerned, the results showed that the male participants were more positive toward their coaches’ democratic behavior than the females. These results provided support for Chelladurai and Saleh (1980) that athletes’ characteristics and situational factors determine coaching behavior preference.

Furthermore, it was found that the athletes’ perceptions of their coaches’ social support behaviors did not differ significantly by gender in team sports. Regarding individual sports, on the other hand, the perceptions of the male athletes’ perceptions of their coaches’ social support behaviors were found to be more positive than those of their female counterparts. Social support behavior encompasses coaching behaviors as to building warm relations between team members, establishing positive atmosphere and working for the health and happiness of each team member.

When the team sports were considered, the perceptions of the female athletes of their coaches’ positive feedback behaviors were more positive than those of the male athletes. Positive feedback refers to the rewarding behavior of the coach. This study suggested that positive feedback behaviors were preferred significantly more by the female athletes of teams than by the athletes of individual sports. On the contrary, the male athletes of individual sports were more positive than their counterparts in terms of the perceptions of positive feedback behaviors.

With regard to age, the results indicated that the perceptions did not change by age. This result went in line with the result obtained by Terry and Howe (1984) that coaching perception was homogeneous regardless of age. On the other hand, this result contradicted Weinberg and Gould’s (2015) result that age influenced athletes’ perceptions of leadership behaviors. More specifically, they argued that as athletes’ grew older, they tended to prefer authoritarian and socially supportive behavior of the coaches more.

## Conclusion

This study attempted to ascertain the perceptions of athletes of their coaches’ leadership behaviors. The results indicated that coaches involved in this study exhibit effective leadership behaviors and that training and instruction was the most perceived leader behavior followed by democratic behavior, positive feedback, social support and autocratic behavior. These results corroborate the results of previous research studies that effective leadership behaviors of sports coaches are associated with success in sport contexts. This is an expected result since the participants are all champion athletes. The participants in this study have a collectivist culture and as evidenced by previous studies in such cultures athletes value training and instruction behavior of their coaches more than anything else. Hence, it is an expected result. In the light of these results, it can be posed that these results of the study support the results of the previous studies in the relevant literature. The results also suggest that gender and type of sport affect perceptions of leadership behaviors. These results endorse the results of previous research as well. Regarding age, there are clashing results in the literature. This study finds that age does not influence athletes’ perceptions of leadership behaviors. In terms of the type of sport, athletes of team sports have more positive perceptions of the training and instruction behavior of the coaches than individual athletes. This result is a novel finding which is expected to move the body of scientific knowledge on the relevant literature forward. The other novel contribution of this study to the existing knowledge on the leadership literature on sports is through the result that gender influences the perceptions of the athletes of team and individual sports regarding democratic, social support and positive feedback behaviors, which calls a need for future studies to investigate such demographic variables in team and individual sports.

Although this study fulfilled the research aim and answered the research questions, there are a few limitations. This study focused on athletes’ perspectives only. Future studies can incorporate both coaches’ and athletes’ perspectives in a single study to evaluate how the self-perceptions of coaches and athletes’ perceptions of their coaches go in line or differ. Another limitation is that the current study delved into the effects of athletes’ demographic variables on the perceptions of coaches’ leadership behaviors since the main focus was athletes. It is recommended that the effect of coaches’ demographic variables should be under the investigation of further studies.

## Data availability statement

The original contributions presented in the study are included in the article/supplementary material, further inquiries can be directed to the corresponding author.

## Ethics statement

The studies involving human participants were reviewed and approved by The Ethics Review Board of the University of Kyrenia. Written informed consent to participate in this study was provided by the participants’ legal guardian/next of kin.

## Author contributions

All authors listed have made a substantial, direct, and intellectual contribution to the work and approved it for publication.

## Conflict of interest

The authors declare that the research was conducted in the absence of any commercial or financial relationships that could be construed as a potential conflict of interest.

## Publisher’s note

All claims expressed in this article are solely those of the authors and do not necessarily represent those of their affiliated organizations, or those of the publisher, the editors and the reviewers. Any product that may be evaluated in this article, or claim that may be made by its manufacturer, is not guaranteed or endorsed by the publisher.
